# Tumor heterogeneity and acquired drug resistance in FGFR2-fusion-positive cholangiocarcinoma through rapid research autopsy

**DOI:** 10.1101/mcs.a004002

**Published:** 2019-08

**Authors:** Melanie A. Krook, Russell Bonneville, Hui-Zi Chen, Julie W. Reeser, Michele R. Wing, Dorrelyn M. Martin, Amy M. Smith, Thuy Dao, Eric Samorodnitsky, Anoosha Paruchuri, Jharna Miya, Kaitlin R. Baker, Lianbo Yu, Cynthia Timmers, Kristin Dittmar, Aharon G. Freud, Patricia Allenby, Sameek Roychowdhury

**Affiliations:** 1 Comprehensive Cancer Center, The Ohio State University, Columbus, Ohio 43210, USA;; 2 Biomedical Sciences Graduate Program, The Ohio State University, Columbus, Ohio 43210, USA;; 3 Division of Medical Oncology, Department of Internal Medicine, The Ohio State University, Columbus, Ohio 43210, USA;; 4 Department of Biomedical Informatics, The Ohio State University, Columbus, Ohio 43210, USA;; 5 Department of Radiology, The Ohio State University, Columbus, Ohio 43210, USA;; 6 Department of Pathology, The Ohio State University, Columbus, Ohio 43210, USA

**Keywords:** biliary tract neoplasm

## Abstract

Cholangiocarcinoma is a highly aggressive and lethal malignancy, with limited treatment options available. Recently, FGFR inhibitors have been developed and utilized in FGFR-mutant cholangiocarcinoma; however, resistance often develops and the genomic determinants of resistance are not fully characterized. We completed whole-exome sequencing (WES) of 11 unique tumor samples obtained from a rapid research autopsy on a patient with FGFR-fusion-positive cholangiocarcinoma who initially responded to the pan-FGFR inhibitor, INCB054828. In vitro studies were carried out to characterize the novel FGFR alteration and secondary *FGFR2* mutation identified. Multisite WES and analysis of tumor heterogeneity through subclonal inference identified four genetically distinct cancer cell populations, two of which were only observed after treatment. Additionally, WES revealed an *FGFR2* N549H mutation hypothesized to confer resistance to the FGFR inhibitor INCB054828 in a single tumor sample. This hypothesis was corroborated with in vitro cell-based studies in which cells expressing *FGFR2–CLIP1* fusion were sensitive to INCB054828 (IC_50_ value of 10.16 nM), whereas cells with the addition of the N549H mutation were resistant to INCB054828 (IC_50_ value of 1527.57 nM). Furthermore, the *FGFR2* N549H secondary mutation displayed cross-resistance to other selective FGFR inhibitors, but remained sensitive to the nonselective inhibitor, ponatinib. Rapid research autopsy has the potential to provide unprecedented insights into the clonal evolution of cancer throughout the course of the disease. In this study, we demonstrate the emergence of a drug resistance mutation and characterize the evolution of tumor subclones within a cholangiocarcinoma disease course.

## INTRODUCTION

Cholangiocarcinoma is an aggressive and deadly rare cancer arising from bile duct epithelial cells with a 5-yr overall survival rate of <2% for advanced stage disease (PDQ Adult Treatment Editorial Board 2002; Razumilava and Gores 2014). Most patients with cholangiocarcinoma present with metastatic unresectable cancer, thus precluding curative therapy (Valle et al. 2010; Razumilava and Gores 2014). Given its poor prognosis and limited treatment options beyond first-line chemotherapy, development and optimization of novel therapies for cholangiocarcinoma are urgently needed.

The fibroblast growth factor receptor (FGFR) signaling pathway is aberrantly activated in ∼20% of cases of intrahepatic cholangiocarcinoma through various genomic alterations including point mutations, copy-number amplifications, and gene fusions (Roychowdhury et al. 2011; Wu et al. 2013). Extending beyond cholangiocarcinoma, alterations in the FGFR signaling pathway have been reported in non-small-cell lung carcinoma, endometrial cancer, and urothelial cancer (Roychowdhury et al. 2011; Wu et al. 2013). Currently, several tyrosine kinase inhibitors, covalent and noncovalent, nonselective and selective FGFR inhibitors are being assessed clinically in patients with metastatic cancer and have shown early responses in those patients with metastatic FGFR-mutant cancers (Gozgit et al. 2012; André et al. 2013; Angevin et al. 2013; Tabernero et al. 2015; Paik et al. 2017; Javle et al. 2018). Although genomic alterations in *FGFR* correlated with initial clinical responses to FGFR inhibitors, multiple secondary mutations in *FGFR* and other cellular signaling pathways have been identified in patients after treatment with FGFR inhibitors. Thus, elucidating the various acquired mechanisms of drug resistance to FGFR inhibitors will be critical for the development of new therapies to overcome resistance and improve the outcome of patients with FGFR-mutant cancers.

Tumor heterogeneity has been shown to negatively impact therapeutic response and contribute to treatment resistance in cancer patients, and thus it remains a major impediment to cancer treatment (Dexter and Leith 1986; Heppner and Miller 1989; Bedard et al. 2013; Fisher et al. 2013; Burrell and Swanton 2014). Both genetic and epigenetic mechanisms within the tumor itself as well as changes in the tumor microenvironment can drive the development of tumor heterogeneity (Heng et al. 2009; Junttila and de Sauvage 2013; Meacham and Morrison 2013). Genomic characterization of primary and recurrent/metastatic tumors from the same patient has further demonstrated spatial and temporal intrapatient tumor heterogeneity (ITH) (Bedard et al. 2013). Recent studies have evaluated ITH and clonal evolution through next-generation sequencing (NGS) methods, demonstrating the critical role of these processes in recurrence and development of therapeutic resistance in urothelial carcinoma, renal cell carcinoma, and acute myeloid leukemia (Ding et al. 2012; Gerlinger et al. 2012, 2014; Faltas et al. 2016). Studies like these, however, are limited in cholangiocarcinoma.

To date, the genomic landscape of cholangiocarcinoma has been largely characterized through tumor biopsies and surgical specimens and, therefore, may not accurately reflect the complex and heterogeneous nature of metastatic and drug-resistant disease (Zou et al. 2014; Ruzzenente et al. 2016; Farshidfar et al. 2017; Jusakul et al. 2017). Recently, Goyal et al. (2017) evaluated three patients with FGFR-fusion-positive cholangiocarcinoma who received the FGFR inhibitor BGJ398. Targeted gene panel sequencing using the commercial Guardant360 assay revealed an FGFR2 V564F gatekeeper mutation in plasma circulating tumor DNA (ctDNA) of all three patients and several additional *FGFR2* mutations in two of the patients. One patient consented to rapid research autopsy, and this enabled the procurement of multiple metastatic tumors for genomic profiling with the FoundationOne assay (315-gene panel) to study acquired drug resistance to the drug BGJ398. This study successfully demonstrated the role of acquired mutations in resistance to BGJ398. It also demonstrated heterogeneity at time of autopsy, because eight of 12 tumor samples assessed lacked a secondary mutation in *FGFR2*. However, there are more than 10 FGFR inhibitors in active drug development in clinical trials, and mechanisms of resistance for each of these drugs remain a significant gap in knowledge. Prior research on acquired resistance mutations in *KIT*, *ABL1*, and *ALK* oncogenes with their respective kinase inhibitors demonstrates that cross-resistance and sensitivity for secondary mutations varies widely, and therefore understanding resistance profiles for other FGFR inhibitors will be essential. Further, evaluating additional patients receiving other FGFR inhibitors with an expanded scope of whole exome (more than 20,000 genes) will be critical to characterizing clonal heterogeneity and evolution with FGFR inhibitors.

In the current work, we present a patient with metastatic cholangiocarcinoma harboring a novel *FGFR2–CLIP1* gene fusion who demonstrated a partial response followed by disease progression while on treatment with the FGFR-selective kinase inhibitor, INCB054828. Through rapid research autopsy of this patient and whole-exome sequencing (WES) of his metastatic cancer, we identified four unique tumor subclones and elucidated their evolution from the normal ancestral cell. Furthermore, we identified a posttreatment secondary kinase mutation in *FGFR2* present in a single metastatic tumor sample and characterized its impact on sensitivity to a variety of FGFR inhibitors in vitro. The results of our in vitro drug sensitivity studies suggest that this mutation conferred resistance to INCB054828 in this patient and thus may have potential as a clinically useful biomarker of resistance. Importantly, characterizing tumor heterogeneity and the ability to detect clonal evolution in patients will facilitate approaches to prevent or overcome treatment resistance and disease recurrence.

## RESULTS

### Clinical Course

**Figure 1.**
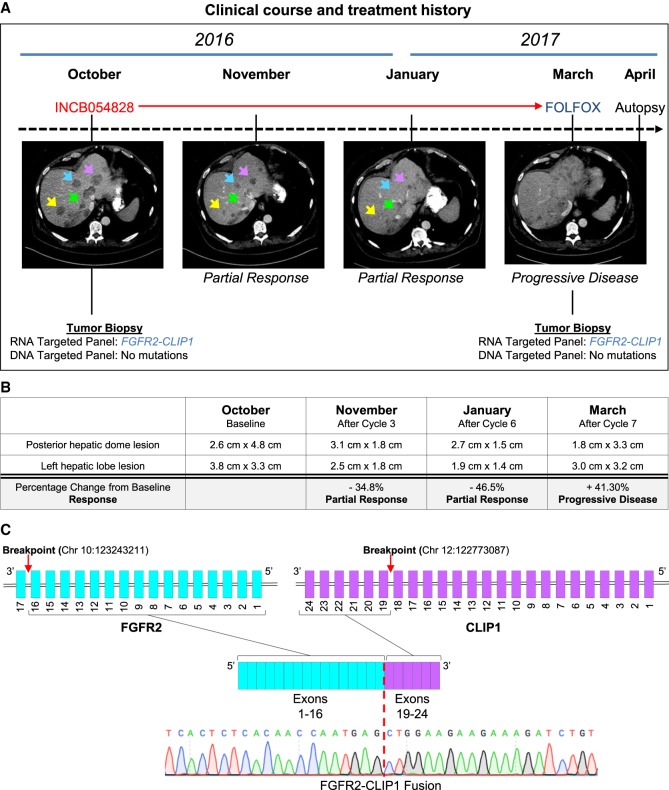
Clinical description of a cholangiocarcinoma patient harboring an *FGFR2–CLIP1* fusion. (*A*) A patient with metastatic cholangiocarcinoma underwent a liver biopsy and sequencing revealed an *FGFR2–CLIP1* gene fusion. He received gemcitabine/cisplatin but after two cycles of therapy had disease progression. He was enrolled on INCB54828 and had a profound radiographic response. After 5.5 mo on therapy, he developed progression and passed away shortly thereafter from his disease. A repeat tumor biopsy at the time of progression did not reveal any secondary mutations. (*B*) Table summarizing two target lesions (posterior hepatic dome lesion and left hepatic lobe lesion) that were tracked throughout the treatment course and had a 34.8% and 46.5% reduction from baseline after cycles 3 and 6, respectively. (*C*) Schematic of the *FGFR2–CLIP1* fusion involving exons 1–16 of *FGFR2* and exons 19–24 of *CLIP1*. Chromatogram traces from Sanger sequencing of the tumor biopsy confirmed the presence of the fusion. Red dashed line indicates breakpoint within the sequence.

A 59-yr-old male presented clinically with abdominal pain and fullness in the fall of 2015. Abdominal CT and MRI scans revealed two small but suspicious-appearing lesions in the liver. He underwent biopsy of one liver lesion, and pathology demonstrated poorly differentiated adenocarcinoma with focal neuroendocrine differentiation (CK7^+^, CDX2^+^, synaptophysin/chromogranin^+^, CK20^−^, TTF1^−^, napsin^−^) consistent with pancreatic or biliary origin. A PET-CT scan showed localized cancer in the right hepatic lobe, and the patient subsequently underwent surgical resection with clear margins and no lymph node involvement. Surgical pathology confirmed intrahepatic cholangiocarcinoma, which was staged as T2aN0. The patient received no adjuvant therapy postsurgery. Five months later, in April 2016, surveillance MRI showed the emergence of new hepatic tumors, prompting palliative treatment with gemcitabine and cisplatin (Gem/Cis). Gemcitabine (1000 mg/m^2^) and cisplatin (25/m^2^) were given on day 1 and day 8 of a 21-d cycle. In June 2016, after two cycles of chemotherapy, CT scans revealed numerous hypodense lesions consistent with worsening of hepatic metastatic disease, and Gem/Cis was stopped. At this time, the patient underwent a repeat tumor biopsy and RNA profiling of his cancer using an NGS assay, OSU-SpARKFuse (Reeser et al. 2017), which revealed a novel gene fusion involving *FGFR2* (exons 1–16) and *CLIP1* (exons 19–24) (Fig. 1A; Table 1). The presence of the fusion was confirmed by reverse transcription PCR and Sanger sequencing with primers designed to flank the breakpoint (Fig. 1C; Supplemental Fig. S1A). CLIP1 is a CAP-Gly domain-containing linker protein 1 that has been shown to regulate the microtubule cytoskeleton. Based on the presence of this novel *FGFR2–CLIP1* fusion in his cancer, at the beginning of October, the patient enrolled in a Phase I/II clinical trial (NCT02393248) evaluating the safety and tolerability of an oral pan-FGFR inhibitor, INCB054828. He received 13.5 mg once daily for days 1–14 per 21-d cycle. Disease assessment after cycles 3 (November) and 6 (January) showed robust partial response by RECIST criteria, consistent with this novel FGFR2 fusion being a driver of his metastatic cancer (Fig. 1A). As part of the study, two target lesions (posterior hepatic dome lesion and left hepatic lobe lesion) were tracked throughout the treatment course and had a 34.8% and 46.5% reduction from baseline after cycles 3 and 6, respectively (Fig. 1B). Prior to starting cycle 8, he was admitted to the hospital with significant weight loss and elevated liver function tests (LFTs), suggesting disease progression. After a total of 5 mo (7 cycles) on INCB054828, CT scans showed a 41.3% increase in size of the two target lesions confirming progressive disease (Fig. 1A,B). At this time, he underwent a repeat postprogression tumor biopsy that confirmed the continued presence of the *FGFR2–CLIP1* fusion (Fig. 1A). One month after receiving the last dose of INCB045828, second-line chemotherapy (FOLFOX) was initiated. He received a single dose of oxaliplatin (190 mg) and fluorouracil (3975 mg). However, he passed away 11 d after receiving this single dose of FOLFOX as a result of liver failure. Prior to passing, he consented to our body donation study for patients with advanced cancer.

**Table 1.** Variant table

Gene	Chromosome	HGVS DNA reference	HGVS protein reference	Variant type	Predicted effect (substitution, deletion, etc.)	dbSNP/dbVar ID	Genotype (heterozygous/homozygous)	ClinVar ID
*FGFR2* and *CLIP1*	RNA Chr 10:123243211 and Chr 12:122773087 DNA Chr 10:123241591 and Chr 12:122782142	N/A	N/A	Chromosomal rearrangement	Likely pathogenic	N/A	N/A	N/A
*FGFR2*	Chr 10:123258036	NM_000141.4, c.1645A>C	N549H	Single-nucleotide variant	Likely pathogenic	rs1057519045	N/A	SUB5631497

### Research Autopsy Reveals Clonal Heterogeneity in Cholangiocarcinoma

**Figure 2.**
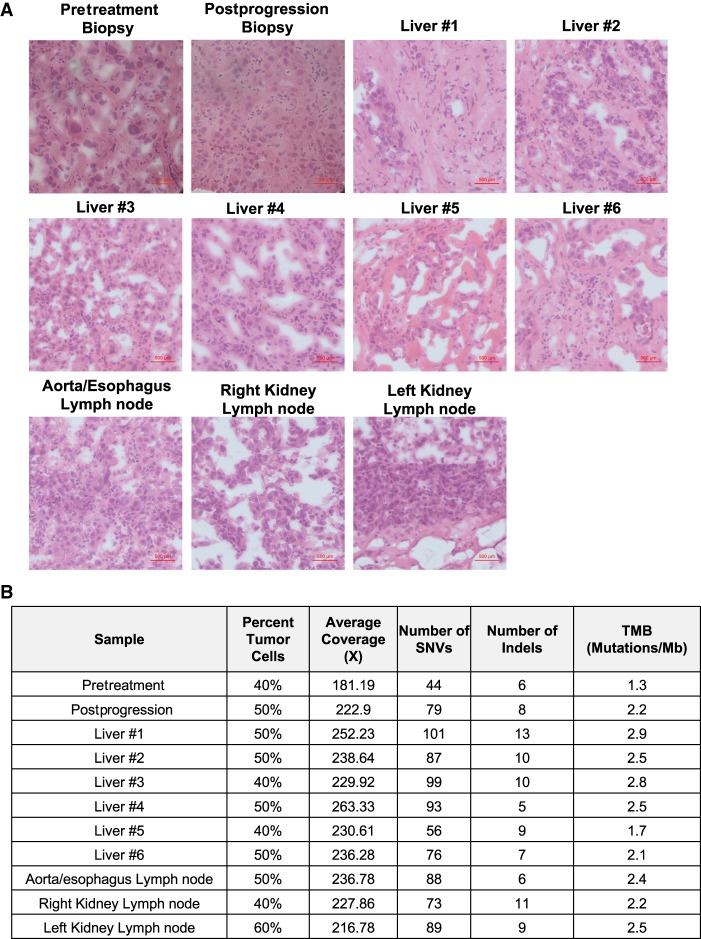
Tumor samples procured at research autopsy. (*A*) Hematoxylin and eosin (H&E) stains of representative slides taken from each tumor sample demonstrating abundant malignant cells. (*B*) Summary of estimated tumor content and WES metrics within each sample. (SNV) Single-nucleotide variant, (TMB) tumor mutational burden.

Upon death of this patient, a research autopsy was performed 8 hours postmortem. Gross examination revealed metastatic tumors involving the liver, omentum, and abdominal and retroperitoneal lymph nodes. Twenty-four liver tumor samples and five separate lymph nodes were procured at the time of autopsy. Although we attempted to sample distinct liver tumors, the patient's liver was predominately cancerous with limited grossly normal liver tissue present (Supplemental Fig. S1B). Samples used for subsequent analysis had at least 40% tumor content as determined by a board-certified pathologist (Fig. 2A,B). In total, a normal blood control and 11 tumor samples (one pretreatment tumor biopsy, one postprogression tumor biopsy, and nine autopsy tumor samples) were chosen for further analysis (Fig. 2B). Sanger sequencing confirmed that the *FGFR2–CLIP1* fusion was present in each tumor sample (data not shown). DNA from these tumors were subjected to WES, yielding 231× average target coverage (Fig. 2B) and revealed a total of 979 somatic variants across all tumors (292 unique somatic variants) (Samorodnitsky et al. 2015a). Two hundred and forty-two of these mutations were unique to the postprogression and autopsy samples (Supplemental File S6). The tumor mutational burden (TMB) of samples ranged from 1.3 mutations/Mb in the pretreatment biopsy to 2.9 mutations/Mb in liver sample #1 (Fig. 2B), consistent with previous studies indicating low TMB in cholangiocarcinoma (Nakamura et al. 2015; Chalmers et al. 2017). All tumor samples were determined to be microsatellite stable (MSS) through analysis of 2539 loci by MANTIS (Kautto et al. 2017). Mutational signatures 16 and 19 were common across tumor samples. Signature 16 has been found in liver cancer and signature 19 has been found in pilocytic astrocytoma, however, their etiologies are unknown (Forbes et al. 2017).

**Figure 3.**
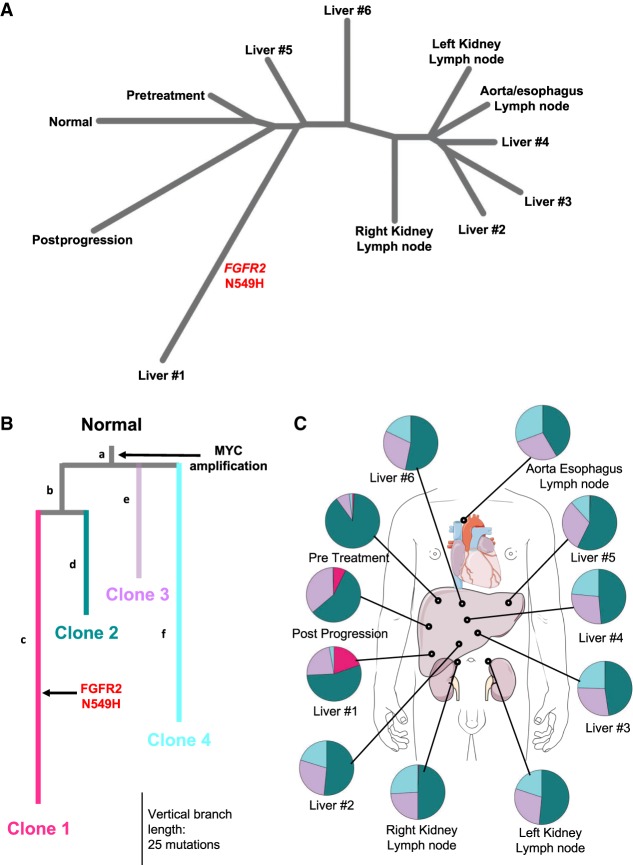
Analysis of tumor heterogeneity. (*A*) Neighbor-joining tree over sets of somatic SNVs in each of 11 tumor samples, with normal defined as the empty set. (*B,C*) Subclonal inference from Canopy. Colors in *B* correspond to subclones in *C*. Letters identify branches of the tree. (*B*) Phylogenetic tree assessment with Canopy revealed four major clonal populations of cells. Each subclone is characterized by a group of mutations. MYC gain was truncal to all subclones. Clone 1 (pink) contained a unique *FGFR2* N549H point mutation. Vertical distance corresponds to increased number of somatic mutations (SNVs and indels). (*C*) Prevalence of four tumor subclones within tumor samples.

The somatic single-nucleotide variants (SNVs) called in each tumor sample were subsequently used to build a phylogenetic tree of tumor samples via the neighbor-joining (NJ) method (Fig. 3A; Saitou and Nei 1987). As expected, the pretreatment sample branched most closely to the normal cells; the two samples are separated by a relatively short genetic distance of 33.2 indicating a high degree of genetic similarity. The postprogression sample had the next closest genetic similarity to the normal, with a genetic distance of 37.6. The liver #1 sample was the most genetically unique tumor sample with a genetic distance of 100.1 from the normal. Liver samples #2, #3, and #4 were clustered with the aorta/esophagus lymph node and left kidney lymph node.

We next utilized Canopy (Jiang et al. 2016) to computationally identify and characterize tumor subclones using both synonymous and nonsynonymous somatic SNVs, CNVs, and indels (Fig. 3B). This analysis revealed four tumor subclones across the 11 samples, with each subclone characterized by a unique group of genomic alterations (Fig. 3B; Supplemental Fig. S2; Supplemental Files S5, S7, S8). A four-clone model was selected because models with additional subclones yielded only marginal increases in BIC (Supplemental Fig. S2). Clones 2 (teal) and 3 (purple) were shared among all samples (Fig. 3C). Clone 4 (cyan) was seen in all except one autopsy sample (liver #1) and was not present in the pretreatment or posttreatment samples (Fig. 3C). Eighty-nine percent of the tumor cells in the pretreatment sample were estimated to be from clone 2 versus ∼40%–60% of the other samples (Fig. 3C). Clone 1 (pink) was primarily found in liver #1 (20%) and at low frequency in the posttreatment sample (7%) (Fig. 3C). This is consistent with the NJ tree, as the relatively large number of mutations unique to clone 1 accounts for the distance of liver #1 and the postprogression samples from all other samples.

Of the 292 distinct mutations (SNVs and indels) identified among these samples, only seven were truncal (i.e., common to all four subclones). Most notable among the truncal events (branch *a*) was a 21.9-Mb gain in Chromosome 8q (Chr 8: 124448804–146364022), containing *MYC* among other genes. Although Canopy did not identify nontruncal mutations shared by clones 3 and 4, post-hoc assignment was permitted to assign mutations to a hypothetical unique common ancestor. No such mutations were assigned, suggesting that clones 3 and 4 diverged relatively early in the tumor's evolution. Of clinical interest, WES revealed an FGFR2 kinase domain mutation, *FGFR2* N549H in a single liver tumor, liver #1 (Fig. 3A). The *FGFR2* N549H mutation occurs in the kinase hinge and has been shown to disengage the molecular breaker resulting in ligand-independent constitutive activation of the FGFR2 kinase (Chen et al. 2007). The *FGFR2* N549H mutation was assigned uniquely to clone 1, which was the most genetically distinct subclone compared to the patient's normal blood DNA (Fig. 3B). Although clone 1 was predicted to be present at low frequency in the postprogression sample, *FGFR2* N549H was not detected in this sample. ddPCR of all samples confirmed that the *FGFR2* N549H mutation was unique to liver #1 (Supplemental Table S1). Of the 111 mutations unique to clone 1, this mutation was estimated to be the 63rd to occur. This led us to hypothesize that the N549H *FGFR2* kinase domain mutation may have been partially responsible for driving resistance to INCB054828 in this patient, occurring along an existing clonal lineage. Driver mutation prediction with CHASM (Carter et al. 2009) predicted only *FGFR2* N549H to be a statistically likely driver (defined as FDR-corrected *P* ≤ 0.05) (Supplemental File S9).

### In Vitro Characterization of Acquired Mutations in the *FGFR2–CLIP1* Fusion and Resistance to the FGFR Inhibitor INCB054828

**Figure 4.**
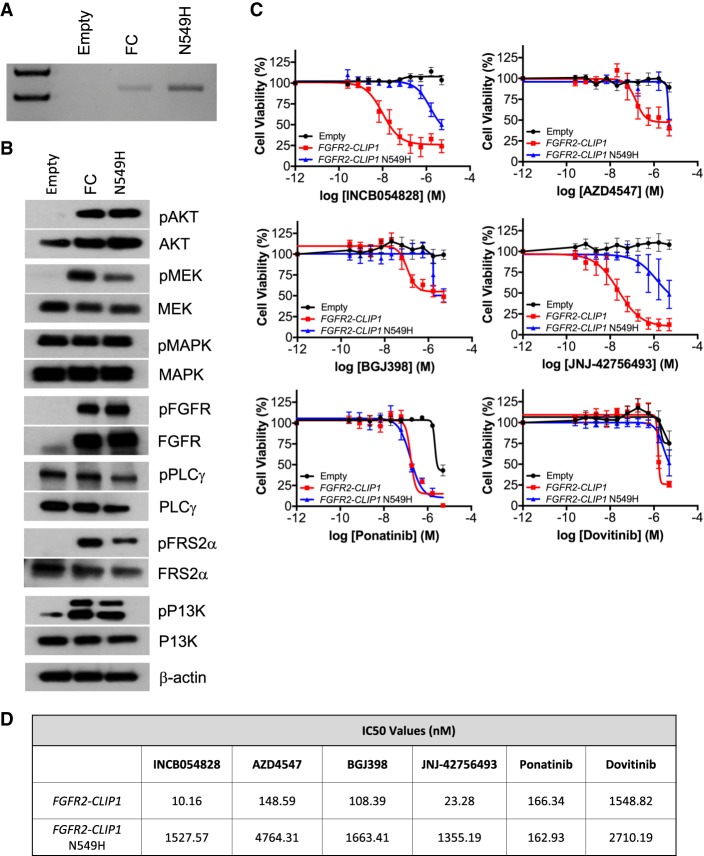
The *FGFR2–CLIP1* is sensitive to FGFR inhibitors, whereas the N549H kinase domain mutation confers resistance. (*A*) RT-PCR confirmed the presence of the *FGFR-CLIP1* fusion in the NIH3T3 *FGFR2–CLIP1* (FC) cells and NIH3T3 *FGFR2–CLIP1* N549H (N549H) cells. The fusion was not detected in the control vector (Empty) transduced cells. (*B*) Total cell lysates from NIH3T3 Empty, FC, and N549H cells were prepared and subjected to western blot analysis with antibodies against pAKT, AKT, pMEK, MEK, pMAPK, MAPK, pFGFR, FGFR, pPLCy, PLC, pFRS2, FRS2, pPI3K, PI3K, and β-actin. (*C*) IC_50_ curves of NIH3T3 Empty, FC, and N549H cells treated with FGFR inhibitors. Data from four replicate experiments are shown. Inhibitors include INCB054828, AZD4547, BGJ398 and JNJ42756493, ponatinib, and dovitinib. (*D*) IC_50_ values are reported for each inhibitor from the curves seen in *C*.

To confirm our clinical findings that the *FGFR2–CLIP1* fusion is exquisitely sensitive to INCB054828 and explore the hypothesis that the *FGFR2* N549H mutation confers resistance to INCB054828, we generated NIH3T3 cells that express either a control (Empty) vector, FGFR2–CLIP1 fusion (FC), or *FGFR2–CLIP1* fusion with the N549H secondary mutation (N549H) and confirmed expression by RT-PCR (Fig. 4A) and Sanger sequencing (data not shown). Western blot analyses of *FGFR2–CLIP1* fusion expression cells demonstrated increases in PI3K/AKT, MAPK/MEK, and FGFR2 signaling pathways with or without N549H (Fig. 4B).

To evaluate the in vitro sensitivity of cells with the *FGFR2–CLIP1* fusion and cells with the *FGFR2–CLIP1* N549H to the FGFR inhibitor INCB054828, we treated NIH3T3 Empty, *FGFR2–CLIP1*, and *FGFR2–CLIP1* N549H cells with increasing doses of INCB054828 or vehicle control (DMSO) ranging from 1.0 nM to 5000 nM and assessed cell viability after 72 h. Treatment of NIH3T3 *FGFR2–CLIP1* (FC) cells with INCB054828 demonstrated substantial and reproducible inhibition of cell viability with an IC_50_ value of 10.16 nM (Fig. 4C,D). Consistent with our hypothesis, the *FGFR2–CLIP1* N549H (N549H) cells were resistant to INCB054828 with an IC_50_ value of 1527.57 nM (Fig. 4C,D). Empty vector control cells (Empty) were not sensitive to INCB054828, which is expected as these cells do not express endogenous FGF ligands or FGF receptors (Fig. 4C). Thus, these data help explain this patient's clinical course with his initial *FGFR2–CLIP1* fusion expressing tumor responding to INCB054828 followed by acquisition of resistance via the N549H mutation.

We subsequently extended these in vitro studies to include additional FGFR inhibitors that are currently being evaluated clinically in patients with metastatic cancer and have shown early responses in patients with FGFR-mutant cancers. AZD4547, BGJ398, and JNJ-42756493 are selective FGFR inhibitors, whereas ponatinib and dovitinib are nonspecific tyrosine kinase inhibitors that target BCR-ABL, VEGFR, PDGFR, SRC, RET, KIT, and FLT1 in addition to FGFR. Our results demonstrated that *FGFR2–CLIP1* cells were sensitive to AZD4547, BGJ398, JNJ-42756493, and ponatinib with IC_50_ values of 148.59 nM, 108.39 nM, 23.28 nM, and 166.34 nM, respectively (Fig. 4C,D). The *FGFR2–CLIP1* N549H cells were less sensitive to BGJ398, AZD4547, and JNJ-42756493, as demonstrated by higher IC_50_ values than the fusion alone (Fig. 4C,D). Interestingly, *FGFR2–CLIP1* N549H cells demonstrated a similar sensitivity to ponatinib as cells with the fusion alone (Fig. 4C,D). Dovitinib was largely ineffective against *FGFR2–CLIP1* without or with the secondary mutation (Fig. 4C,D). Empty vector control cells (Empty) were not sensitive to AZD-4547, BGJ398, or JNJ-42756493 (Fig. 4C,D). However, at the highest dose (5 µM) of ponatinib and dovitinib, the control cells (Empty) demonstrated decreased cell viability, which was not surprising as ponatinib and dovitinib are nonspecific inhibitors of FGFR (Fig. 4C,D). Taken together, these data demonstrate that the *FGFR2–CLIP1* fusion confers sensitivity to some, but not all, FGFR inhibitors. The *FGFR2* N549H secondary mutation confers resistance to most FGFR inhibitors, but ponatinib could be used to overcome this acquired drug resistance.

## DISCUSSION

Tumor heterogeneity has been shown to have a critical role in response to therapy, development of resistance, and clinical outcome in patients with cancer (Rottenberg et al. 2012; Choi et al. 2017; Joung et al. 2017). Rapid research autopsy has emerged as a powerful strategy to study tumor heterogeneity, as it enables essentially unlimited sampling of all sites of metastatic disease throughout the body that would otherwise not be feasible through surgical resections or tumor biopsies (Krook et al. 2019). A number of recent studies have utilized rapid research autopsy to characterize tumor heterogeneity, clonal evolution, and mechanisms of acquired therapeutic resistance in breast, urothelial, pancreatic, and colorectal cancer. For instance, Saito et al. utilized research autopsy in breast cancer to assess trastuzumab resistance in primary versus metastatic sites (Saito et al. 2015). Faltas et al. (2016) performed rapid autopsy of two patients to construct phylogenetic trees of urothelial carcinoma. Here, we present our findings from rapid research autopsy of a patient with metastatic cholangiocarcinoma. This is the first study to evaluate clonal heterogeneity based on exome sequencing in cholangiocarcinoma, as well as the first description of acquired resistance to INCB54828, an oral FGFR inhibitor.

Most previous and current autopsy studies utilize methods such as clonal ordering (Merlo et al. 2006) and NJ (Saitou and Nei 1987) to identify and quantify relationships between different tumor regions and/or sites of metastatic cancer. In this study, we utilized the NJ method to generate a tumor-centric tree to assess similarities and differences among the pretreatment biopsy, postprogression biopsy, and nine unique tumors collected at the time of autopsy. The NJ analysis showed that the liver #1 sample with its unique *FGFR2* N549H point mutation is an outlier versus the other liver samples from autopsy. NJ and related phylogenetic methods are powerful tools to assess high-level relatedness among tumors and identify exceptional tumors; however, they cannot capture the clonal heterogeneity present within discrete tumor masses or cross-seeding between sites.

Analysis of clonal evolution continues to develop as technical and computational challenges and the limited availability of large-scale autopsy data are overcome. In addition to NJ, we performed subclonal inference using Canopy (Jiang et al. 2016), which revealed four genetically distinct tumor subclones. Of these subclones, three were dominant across all 11 samples, with each subclone characterized by a specific set of mutations (Fig. 2B,C). The *FGFR2* N549H mutation in clone 1 was unique to a single liver sample despite our in vitro data confirming its role as a resistance mutation. This pattern of site-unique *FGFR2* resistance mutations was previously observed by Goyal et al. (2017) in which only four of 12 distinct metastatic autopsy samples were found to have acquired secondary mutations in FGFR2 serving to bypass the FGFR inhibitor effect. Each of these samples harbored unique FGFR mutations (K641R and N549H) with only one sample having two FGFR mutations (E565A and K641R). Meanwhile, the remaining eight sites were wild type (wt) for FGFR2. The observations seen by Goyal et al. along with our work presented here suggest that multiple independent drug resistance mechanisms, including FGFR-independent mechanisms, are likely contributing to tumor progression. Interestingly, in our model, clone 4 was specific to tumor samples collected at the time of autopsy, suggesting that either the biopsies missed a population of cells or that this subclone developed after the posttreatment biopsy. FOLFOX was administered after the collection of the posttreatment biopsy, but as this patient only received one dose of FOLFOX before passing away soon afterward, we do not believe that this single dose substantially affected the heterogeneity present at the time of autopsy. These findings provide evidence for the presumed notion that tumor biopsies do not accurately reflect the full complexity and heterogeneous nature of the disease. Clones 2–4 were seen in all autopsy samples at similar proportions. As the liver tumors were largely confluent at the time of autopsy (Supplemental Fig. S1B), multiple samples may have come from the same tumor. Another possibility is metastatic cross-seeding, as was observed by Savas et al. (2016) using their tool superFREQ (Flensburg et al. 2018) for subclonal analysis of four metastatic breast cancer cases, and by Brady et al. (2019) in pediatric osteosarcoma. Co-metastasis of multiple subclonal populations can also explain this distribution, as has been demonstrated to occur in breast ductal carcinoma (Casasent et al. 2018). Clone 2 was substantially reduced in the posttreatment and autopsy samples versus the pretreatment biopsy. One potential explanation is that clone 2 was more sensitive to INCB054828 than clones 1, 3, and 4. In clone 1, the decreased sensitivity is likely due to the *FGFR2* N549H point mutation, evolving from a common lineage as clone 2. Although resistance mechanisms for clones 3 and 4 could not be determined through WES, and driver prediction did not indicate any other likely driver mutations, we hypothesize that there can be multiple independent drug resistance adaptations within a single patient. Previous studies have demonstrated that in addition to secondary kinase domain mutations, activation in the Akt, MAPK, and PTEN pathways can mediate resistance to FGFR inhibition (Datta et al. 2017; Goyal et al. 2017; Malchers et al. 2017). Although there was no evidence for PTEN mutations in this patient, transcriptome sequencing would be needed to assess the activation of other pathways. Studies are ongoing in our laboratory to identify FGFR-independent mechanisms of resistance and to define their contributions clinically, including RNA sequencing.

WES of this patient and derivation of a phylogenetic tree suggests that ancestral genotypes can persist throughout the disease course, despite the evolution of highly derived subclones. We note that only four SNVs, three indels, and one copy-number gain were detected in the trunk of this patient's phylogenetic tree (branch *a*), indicating that development of *FGFR2*-fusion-positive cholangiocarcinoma may only require a small number of other initiating events (Fig. 3B). For instance, clone 4 was only detected in autopsy samples, yet evolved from a distant ancestor to clones 1, 2, and 3. Clone 1 did not directly evolve from clone 2, but rather it shares a common ancestor with clone 2, which must have been extant before treatment (for clone 2 to be found in the pretreatment sample). Such persistent ancestral cells may serve as an “uncommitted” tumor reserve capable of developing new adaptations throughout the disease course. Subclonal analysis, such as in this study, permits the characterization of cancer as a dynamic process of multiple evolving and diverging cellular populations rather than a singular entity in a patient. This view of cancer permits somatic variants, a staple of cancer genomics, to be viewed in a new context. However, phylogeny inference from short-read bulk sequencing has several inherent limitations, most notably that phylogenetic solutions consistent with variant fractions and CNVs are frequently nonunique (Pradhan and El-Kebir 2018). Emerging long-read and single-cell sequencing technologies will permit more certain and accurate modeling of phylogeny by directly assessing the phasing of subclonal mutations.

Lessons learned from studying molecular mechanisms of resistance to ABL, EGFR, ALK, KIT, and RAF inhibitors in human cancers have highlighted the need for next-generation kinase inhibitors that are effective against acquired secondary resistance mutations (Demetri 2011; Roychowdhury and Talpaz 2011; Gainor and Shaw 2013; Lito et al. 2013; Van Allen et al. 2014; Hrustanovic et al. 2015). For example, Friboulet et al. demonstrated that crizotinib-induced resistance mutations in ALK-fusion-positive non-small-cell lung cancer (NSCLC) can be overcome by treatment with ceritinib (Friboulet et al. 2014). Furthermore, mutant-selective allosteric inhibitors have shown promise in overcoming the secondary EGFR resistance mutation T790M in NSCLC following EGFR-directed therapy (Jia et al. 2016). Thus, these studies may inform strategies to overcome secondary resistance mutations to FGFR-targeted therapies as several preclinical studies have demonstrated the emergence of a mutation at the gatekeeper residue or other residues within the ATP-binding pocket as well as other mutations in FGFR1–3 (Chell et al. 2013). Unfortunately, several potent and selective ATP-competitive small molecule FGFR inhibitors currently in clinical trials, including INCB054828, BGJ398, AZD4547, and LY2874455, share structural similarities and are ineffective in overcoming the gatekeeper mutations (Chae et al. 2017). Although not considered a gatekeeper mutation, the *FGFR2* N549H mutation is in the vicinity of the ATP binding pocket. Notably, our in vitro findings provide further support for the cross-resistance of multiple FGFR inhibitors, as cells harboring the secondary FGFR mutation N549H were resistant to INCB054828, AZD4547, BGJ398, JNJ-42756493, and dovitinib. Because of this, there has been interest in the use of structure-based drug design to develop a class of next-generation inhibitors that would overcome resistance mutations located in the FGFR2 ATP-binding pocket (Tan et al. 2014). Interestingly, we demonstrated that *FGFR2* N549H retained sensitivity to ponatinib. The clinical use of ponatinib in this context is supported by pharmacokinetic data in patients demonstrating a steady state ponatinib plasma concentration of 145 nM attained 4–8 h after receiving the maximum approved dose of 45 mg (Cortes et al. 2012). Unfortunately, there are serious adverse cardiovascular events associated with ponatinib, which are often dose-limiting (Gagnieu et al. 2013). Thus, the development of next-generation FGFR inhibitors has the potential to dramatically impact the clinical care of patients receiving FGFR-targeted therapies.

In summary, this work suggests that clonal heterogeneity contributes to acquired clinical resistance to the novel FGFR inhibitor, INCB054828, in cholangiocarcinoma. Although limited to a single patient, this is the first study, to our knowledge, to define a mechanism of acquired resistance to INCB054282 through a secondary mutation to the FGFR inhibitor, INCB054828. Through rapid research autopsy and WES, we determine the presence of four tumor subclones and elucidate their evolution in metastatic tissues over time in a patient with FGFR2-fusion-positive cholangiocarcinoma. Furthermore, we identified a posttreatment secondary kinase mutation in FGFR2, present in a single metastatic tumor sample demonstrating the significance of intertumor heterogeneity within the same patient. We characterized the impact of the N549H mutation on sensitivity to different FGFR inhibitors in vitro. The results of our in vitro drug sensitivity studies suggest that this mutation conferred resistance to INCB054828 in this patient and thus may have potential as a clinically useful biomarker of resistance. Overall, our findings suggest that secondary FGFR mutations are drivers of acquired clinical resistance. Understanding these mechanisms of resistance along with FGFR kinase domain-independent mechanisms of resistance will facilitate approaches to prevent or overcome treatment resistance and disease recurrence and guide clinical strategies for these patients.

## METHODS

### Research Autopsy and Patient Samples

The patient consented to an IRB-approved study for high-throughput sequencing of tumor and normal specimens (OSU-13053, NCT02090530) at the James Cancer Hospital and The Ohio State University. OSU-SpARKFuse, a targeted RNA-based NGS assay to detect gene fusions, and a targeted DNA sequencing assay to detect single-nucleotide variations were performed on tumor biopsy specimens as previously described (Reeser et al. 2017). The patient also consented to a body donation study. Upon death of this patient, next of kin informed the research team, who arranged for transportation to the OSU Regional Autopsy Center, and the autopsy was performed 8-h postmortem. Guided by radiographic scans, all visible malignant as well as adjacent normal tissues were collected and frozen in optimal cutting temperature (OCT) compound. Following the autopsy, the patient was returned to the funeral home.

### Whole-Exome Sequencing

Genomic DNA was extracted from frozen tumor biopsy samples and tumors collected during autopsy using the QIAamp DNA Mini Kit. The QIAamp DNA Mini Blood Kit was used to extract genomic DNA from blood. WES was performed as described below. Briefly, the KAPA Hyper Prep Kit (Roche) was used for library preparation, and libraries were enriched using the xGEN Exome Research Panel v1.0 from Integrated DNA Technologies. 2 × 150-bp paired-end sequencing was performed on an Illumina HiSeq4000 at The Genomics Services Laboratory at Nationwide Children's Hospital (Columbus, Ohio).

### Histology

Freshly collected tumor biopsy and autopsy samples were immediately embedded and frozen in OCT compound (Fisher). Frozen sections were cut from tumor biopsy and autopsy samples at 5 µm on a Leica Cryostat CM1950 for H&E staining. A board-certified pathologist reviewed representative slides for each tumor block for estimated tumor content.

### Bioinformatics Analysis

All bioinformatics analyses were performed using the Oakley supercomputer at the Ohio Supercomputer Center (“Oakley supercomputer” 2012). Alignment of WES data to the human genome version hg19 was performed with Burrows–Wheeler Aligner (bwa) (Li and Durbin 2009) version 0.7.14. Duplicate reads were removed using Picard (“Picard Tools - By Broad Institute”) version 2.3.0. Picard and GATK (McKenna et al. 2010) version 3.5 were used to perform quality recalibration and local realignment around indels. SNV and indel calling were performed with VarScan2 (Koboldt et al. 2012) version 2.3.9 and bam-readcount (Larson and Abbott 2016) as previously described (Chen et al. 2019; Supplemental File S1). SNVs and indels were annotated using ANNOVAR (Wang et al. 2010) (revision #11f4bb, 2016-02-01). Putative driver mutation analysis was performed with CRAVAT (Douville et al. 2013) version 5.2.4, using the CHASM (Carter et al. 2009), algorithm version 3.1. TMB was computed as the sum of all called somatic SNVs and indels within the capture region in each sample, divided by the size of the capture region (∼38.9 Mb). Microsatellite instability (MSI) testing was performed with MANTIS (Kautto et al. 2017) using the recommended threshold of 0.4 to call MSI and a set of 2539 whole-exome microsatellite loci (Bonneville et al. 2017). Mutational signatures were called with deconstructSigs (Rosenthal et al. 2016) version 1.8.0 using the COSMIC Mutational Signatures set (Forbes et al. 2017), exome2genome trinucleotide frequency correction, and otherwise default settings (Supplemental File S2).

CNV calling was performed using FALCON (Chen et al. 2015) version 0.2, utilizing germline tumor and normal variants (Supplemental File S3). The QC procedure provided with Canopy (Jiang et al. 2016) was used to reduce false-positive segmentations, with default length and ΔCN settings. For each sample, rdep (read depth ratio) was the ratio of aligned reads in tumor versus normal. FALCON was initially run with threshold 0.3. Resulting CNVs were manually curated to identify genomic regions with major copy number >2 or minor copy number <0.5 in at least one sample. For each curated region, a common pair of breakpoints was estimated across all tumors, and FALCON was rerun with threshold 0.2 and ${\hat{\tau }}_{chr}$ set to the nearest SNPs to each breakpoint in the chromosome (Supplemental File S4). Matrices **W**_M_, **W**_m_, **ε**_M_, and **ε**_m_ (used for Canopy input) were obtained from FALCON output, and matrix **Y** was determined by calculating the overlap of mutations used for tree building with curated CNV regions.

To generate a sample-based phylogenetic tree, a distance matrix was first computed as follows:

$$d_{ij}=|S_{i}\;\Delta \;S_{j}|,$$

where $D\in Z^{\left(N+1\right)\times \left(N+1\right)}$ is the distance matrix, *N* is the number of samples, *S*_*i*_ is the set of somatic SNVs called in sample *i* ∈ 1,…,*N*, and Δ is the set symmetric difference. The set of somatic SNVs in normal, corresponding to *i* = *N* + 1, is the empty set (by definition); therefore, the distance between normal and any tumor collapses to the number of SNVs in that tumor. The tree was generated over **D** via NJ (Saitou and Nei 1987) with RapidNJ (Simonsen et al. 2008) version 2.3.2 and visualized using Interactive Tree of Life (iTOL) (Letunic and Bork 2016) version 4.2.3. Normal is regarded as the root of the tree. Subclonal-based phylogenetic analysis with Canopy and maximum likelihood-based post-hoc assignment of somatic SNVs and indels to the resulting tree was performed as previously described (Chen et al. 2019). Canopy computes a Bayesian information criterion (BIC; Schwarz 1978) score for each potential number of subclones, which was used to determine the number of subclones that best represents the data. Note that Canopy estimates its normal cell fractions based on variant fractions and CNV data, and reported purities can differ from pathologist estimates, likely because different sections of the tumor blocks were sequenced than were reviewed by the pathologist (Supplemental File S5).

Mutations within each branch of the tree were temporally ordered using a Bradley–Terry model (Bradley and Terry 1952). For any tree edge *z* ∈ *Z*, we have a set of mutations *V*_*z*_. For convenience, define VAF_*i*_(*v*) as the VAF of mutation *v* in sample *i* ∈ 1,…,*N*. Given variants *v*_1_, *v*_2_ ∈ *V*_*z*_, we compute the score of *v*_1_ versus *v*_2_ in *i* as follows:

$$w_{i}(v_{1},v_{2})=\{\begin{array}{cc} \;1, & \mathrm{VA}F_{i}(v_{1})>\mathrm{VA}F_{i}(v_{2}),\;\\ \;0, & \mathrm{VA}F_{i}(v_{1})<\mathrm{VA}F_{i}(v_{2}),\;\\ 0.5, & \mathrm{VA}F_{i}(v_{1})=\mathrm{VA}F_{i}(v_{2}).\; \end{array}$$

We now define $W\in R^{\left|V_{z}\left|\times \right|V_{z}\right|}$, the wins matrix, over all samples:

$$W_{\;jk}=\{\begin{array}{rl} & \overset{N}{\underset{i=1}{\sum }}w_{i}(v_{j},\;v_{k}),\;\;j\neq k,\;\\ & 0,\;\;\;\;\;\;\;\;\;j=k,\; \end{array}\;j,\;k\in 1,\ldots ,|V_{z}|.$$

We utilized the R package BradleyTerryScalable (Kaye and Firth 2017) version 0.1.0 with *a* = 1.1, which implements the maximum a priori estimate of Caron and Doucet (Caron and Doucet 2012). Results are returned as abilities, such that

$$P(v_{1}\;\mathrm{occurred}\;\mathrm{before}\;v_{2})=\frac{\pi_{1}}{\pi_{1}+\pi_{2}},$$

where *π*_1_ and *π*_2_ are the abilities of mutations *v*_1_ and *v*_2_. Note that ability scores denote confidence in ordering, not the time intervals between acquisition of mutations. This analysis was performed independently for each edge of the clonal phylogeny tree, utilizing both mutations supplied to Canopy and those retroactively assigned to the tree.

### Droplet Digital PCR and Analysis

Isolated genomic DNA was amplified using a custom-designed probe for the *FGFR2* N549H point mutation (PrimePCR ddPCR Mutation Assay, Bio-Rad) and the ddPCR Supermix for Probes (Bio-Rad). The reaction mixture consisted of 250 ng of DNA template (8 µL), 10 µL of ddPCR Supermix for Probes (Bio-Rad), and 2 µL of the primer/probe mixture. Droplets were generated using the QX200 Droplet Generator (Bio-Rad) and then transferred to a 96-well plate (Eppendorf) for PCR amplification with the following conditions: 5 min at 95°C, 40 cycles of 94°C for 30 sec, 55°C for 1 min, followed by 98°C for 10 min (ramp rate 2°C/sec). Droplets were analyzed with the QX200 Droplet Reader (Bio-Rad) for fluorescent measurement of FAM and HEX probes. Gating was performed based on positive and negative controls, and mutant populations were identified. All reactions were run in duplicate. The ddPCR data were analyzed with QuantaSoft analysis software (Bio-Rad) to obtain fractional abundance of the mutant DNA alleles in the wt/normal background.

### cDNA Plasmid Generation, Lentivirus Production, and Transduction

The *FGFR2–CLIP1* fusion was produced and cloned into the pLVX-IRES-Puro vector (Clontech) by GenScript (Supplemental Fig. S1). Using site-directed mutagenesis, the *FGFR2* N549H mutation was introduced into the fusion by GenScript. NIH3T3 cells were stably transduced with either empty, *FGFR2–CLIP1* or *FGFR2–CLIP1* N549H lentiviral vectors. Cells were selected in puromycin (1 µg/ml; Sigma) for 72 h prior to their use in downstream experiments.

### RNA Isolation, RT-PCR, and Sanger Sequencing

RNA was isolated from cell lines, and cDNA was synthesized using the Quick-RNA MiniPrep Kit (Zymo) and the iScript cDNA Synthesis Kit (Bio-Rad), respectively. cDNA was subsequently PCR amplified with *FGFR2–CLIP1* and *FGFR2* N549H fusion specific primers (IDT). Primer sequences are listed in Table 2. The PureLink Quick PCR Purification Kit (Invitrogen) was used to purify amplified PCR product and samples were then Sanger sequenced (The Ohio State University Comprehensive Cancer Center Genomics Shared Resource, Columbus, OH).

**Table 2.** Primer sequences

Target	Forward	Reverse	Product size (bp)
FGFR2–CLIP1	5′-CAGAGACCAACGTTCAAGCA-3′	5′-CGGCATCCTTTTCTGTGAGT-3′	214
N549H	5′-GTGGCCGTGAAGATGTTGAA-3′	5′-AGGTATTCTCGGAGGTTGCC-3′	188

Primer sequences used for PCR and Sanger sequencing to confirm the presence of either the fusion or the mutation.

### Cell Culture

NIH3T3 and HEK293T cell lines were purchased from American Type Culture Collection (ATCC) and cultured in a humidified incubator at 37°C and 5% CO_2_. Cells were cultured according to the ATCC-recommended protocols. All cell lines were routinely subjected to short tandem repeat profiling to confirm identities and mycoplasma testing using the e-Myco plus Mycoplasma PCR Detection Kit (Bulldog Bio).

### Western Blotting

Western blot assays were carried out using established protocols and probed with the following antibodies: phospho-Akt (Ser473) 1:1000 (Cell Signaling 9271), Total Akt 1:1000 (Cell Signaling 9272), phospho-MEK1/2 1:5000 (Cell Signaling 9154), Total MEK1/2 1:5000 (Cell Signaling 9122), p44/42 MAPK (Erk1/2) 1:5000 (Cell Signaling 9101), Total MAPK 1:5000 (Cell Signaling 9102), phospho-FGF Receptor (Tyr653/654) 1:500 (Cell Signaling 3471), FGF Receptor 2 (D4L2V) 1:500 (Cell Signaling 23328), phospho-PLCγ1 (Tyr783) 1:1000 (Cell Signaling 14008), PLCγ1 (D9H10) 1:1000 (Cell Signaling 5690), phospho-FRS2-α (Tyr196) 1:1000 (Cell Signaling 3864), FRS2 1:1000 (abcam 10425), phospho-PI3 Kinase p85 (Tyr458)/p55 (Tyr199) 1:1000 (Cell Signaling 4228), PI3 Kinase p85 (19H8) 1:000 (Cell Signaling 4257), β-actin 1:10000 (Cell Signaling 4967).

### Drug Sensitivity Assays

NIH3T3 Empty, *FGFR2–CLIP1*, *FGFR2–CLIP1* N549H cells were plated at a density of 10,000 cells per well in 96-well plates. Cells were treated for 72 h with either INCB054828 (Incyte), BGJ398 (Cayman Chemical), JNJ-42756493 (Cayman Chemical), AZD-4547 (Cayman Chemical), ponatinib (Cayman Chemical), or dovitinib (Cayman Chemical) ranging from 0.01 to 5000 nM. Quantification of viable cells was assessed using an MTS/PMS colorimetric assay. IC_50_ values were calculated in Prism (GraphPad) using a four-parameter dose–response model.

## ADDITIONAL INFORMATION

### Data Deposition and Access

Data used for the analyses presented in the manuscript have been submitted to dbGaP (https://ncbi.nlm.nih.gov/gap) under the project accession number phs001830.v1.p1. The *FGFR2-CLIP1* fusion gene variant and the secondary *FGFR2* mutation identified in the patient have also been deposited to ClinVar (https://ncbi.nlm.nih.gov/clinvar/) under the accession numbers SCV000927106 and SCV000914229.1.

### Ethics Statement

The patient consented to an IRB-approved study for high-throughput sequencing of tumor and normal specimens (OSU-13053, NCT02090530) at the James Cancer Hospital and The Ohio State University. OSU-SpARKFuse, a targeted RNA-based next-generation sequencing assay to detect gene fusions, and a targeted DNA sequencing assay to detect single-nucleotide variations were performed on tumor biopsy specimens as previously described (Samorodnitsky et al. 2015b; Reeser et al. 2017). The patient also consented to a body donation study.

### Acknowledgments

We thank Jenny Badillo for her administrative support. We also thank current and past members of the Roychowdhury Precision Cancer Medicine Team, The Ohio State Comprehensive Cancer Center, and James Cancer Hospital and Pelotonia for its community support. We most importantly thank this patient and his family. We thank the Ohio Supercomputer Center for computing resources.

### Author Contributions

M.A.K. and K.R.B. were involved in the generation of in vitro data. C.T. assisted with ddPCR experiments and analyses. A.M.S., T.D., and D.M.M. processed and prepared samples for next-generation sequencing. Computational analyses and interpretation were performed by R.B., A.P., E.S., and J.M. L.Y. supervised statistical analyses. P.A. supervised the rapid research autopsy. M.A.K., R.B., H.-Z.C., J.W.R., and M.R.W. completed dissection, collection, and processing of autopsy tumor samples. A.G.F. analyzed tumor samples for quality and tumor cell content. H.-Z.C., J.W.R., M.R.W., and S.R. received informed consent for the body donation and autopsy study. K.D. performed tumor biopsies, and S.R. treated this patient. M.A.K., R.B., and S.R. wrote, reviewed, and revised the manuscript. All authors have read and approved the final manuscript.

### Funding

S.R. has received support from an American Cancer Society grant MRSG-12-194-01-TBG, the Prostate Cancer Foundation, NCI UH2CA202971 (OSU-SpARKFuse), NCI UH2CA216432 (MSIDx), American Lung Association, and Pelotonia. M.A.K. was in part supported by a T32 Oncology Training Grant (5T32CA009338) and Award Number Grant TL1TR002735 from the National Center for Advancing Translational Sciences. R.B. was in part supported by a T32 T32GM068412 and a Pelotonia graduate student fellowship. H.-Z.C. was supported by a Pelotonia postdoctoral fellowship and an ASCO Conquer Cancer Foundation Young Investigator Award. M.R.W. was supported by the Helene Fuld Health Trust Nursing Scholarship. Incyte Corporation provided INCB054828 for in vitro characterization but no funding was provided.

### Competing Interest Statement

S.R. participated in Advisory Boards for Incyte Corporation (2017), AbbVie, Inc. (2017), and QED Therapeutics (2018). S.R. received honoraria from IDT Integrated DNA Technologies (2017) and Illumina (2018). Incyte Corporation provided INCB054828 for in vitro characterization but no funding was provided.

## Supplementary Material

Supplemental Material
