# Supplementary material for: Tumor heterogeneity and acquired drug resistance in FGFR2-fusion-positive cholangiocarcinoma through rapid research autopsy
Source: Cold Spring Harb Mol Case Stud. 2019 Aug;5(4):a004002. doi: 10.1101/mcs.a004002 (PMC6672025; doi:10.1101/mcs.a004002)
Supplement: Supplemental Material [file supp_5_4_a004002__index.html]

Supplemental Material 

# Tumor heterogeneity and acquired drug resistance in FGFR2-fusion-positive cholangiocarcinoma through rapid research autopsy

## Supplemental Material

- Supplemental\_Figure1.pdf
- Supplemental\_Figure2.pdf
- Supplemental\_S1.xlsx
- Supplemental\_S2.xlsx
- Supplemental\_S3.xlsx
- Supplemental\_S4.xlsx
- Supplemental\_S5.xlsx
- Supplemental\_S6.xlsx
- Supplemental\_S7.xlsx
- Supplemental\_S8.xlsx
- Supplemental\_S9.xlsx
- Supplemental\_Table1.pdf
