# Supplementary material for: Tumor heterogeneity and acquired drug resistance in FGFR2-fusion-positive cholangiocarcinoma through rapid research autopsy
Source: Cold Spring Harb Mol Case Stud. 2019 Aug;5(4):a004002. doi: 10.1101/mcs.a004002 (PMC6672025; doi:10.1101/mcs.a004002)
Supplement: Supplemental Material [file supp_mcs.a004002_Supplemental_Figure1.pdf]

Supplemental Figure 1

A

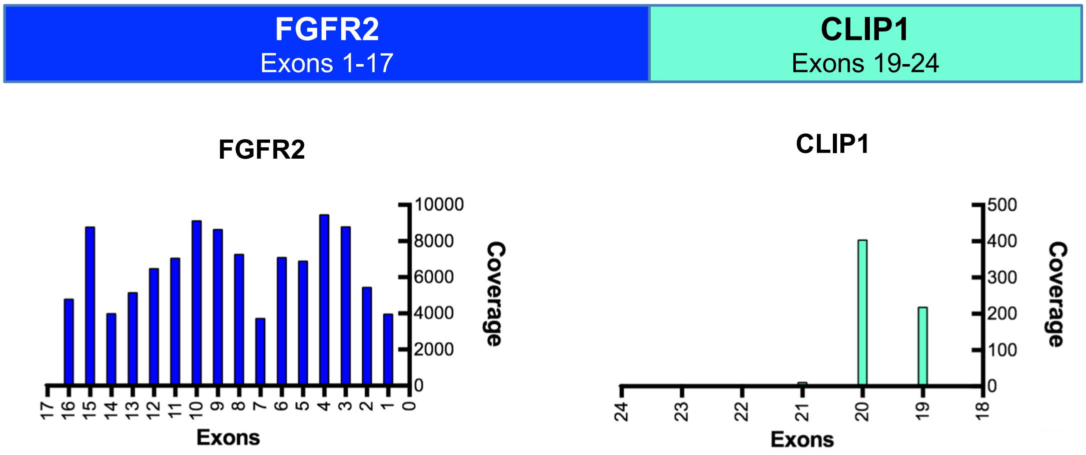

B

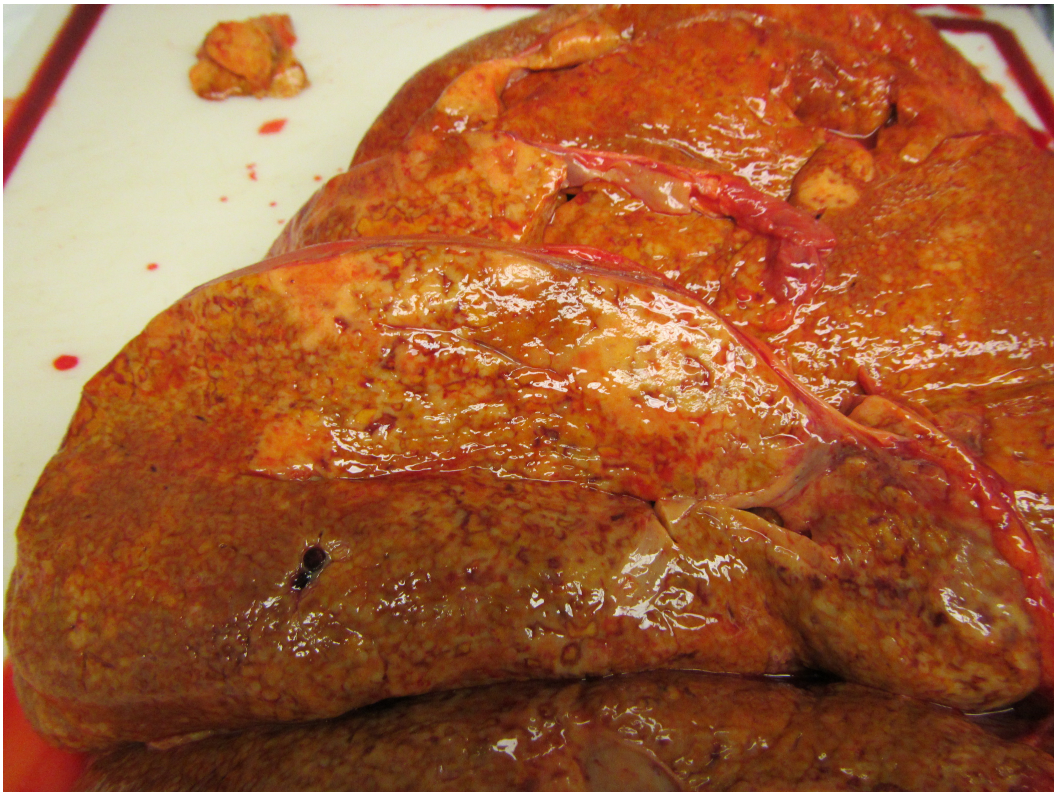

Supplemental Figure 1. Coverage of novel FGFR2 fusion detected and gross liver image. A. A novel fusion containing exons 1-17 of *FGFR2* and exons 19-24 of *CLIP1* was detected in a patient with cholangiocarcinoma. Bar graphs represent average exon read depth for *FGFR2* and *CLIP1* exons. B. Gross image of the liver at the time of autopsy.
