# Supplementary material for: Tumor heterogeneity and acquired drug resistance in FGFR2-fusion-positive cholangiocarcinoma through rapid research autopsy
Source: Cold Spring Harb Mol Case Stud. 2019 Aug;5(4):a004002. doi: 10.1101/mcs.a004002 (PMC6672025; doi:10.1101/mcs.a004002)
Supplement: Supplemental Material [file supp_mcs.a004002_Supplemental_Figure2.pdf]

Supplemental Figure 2

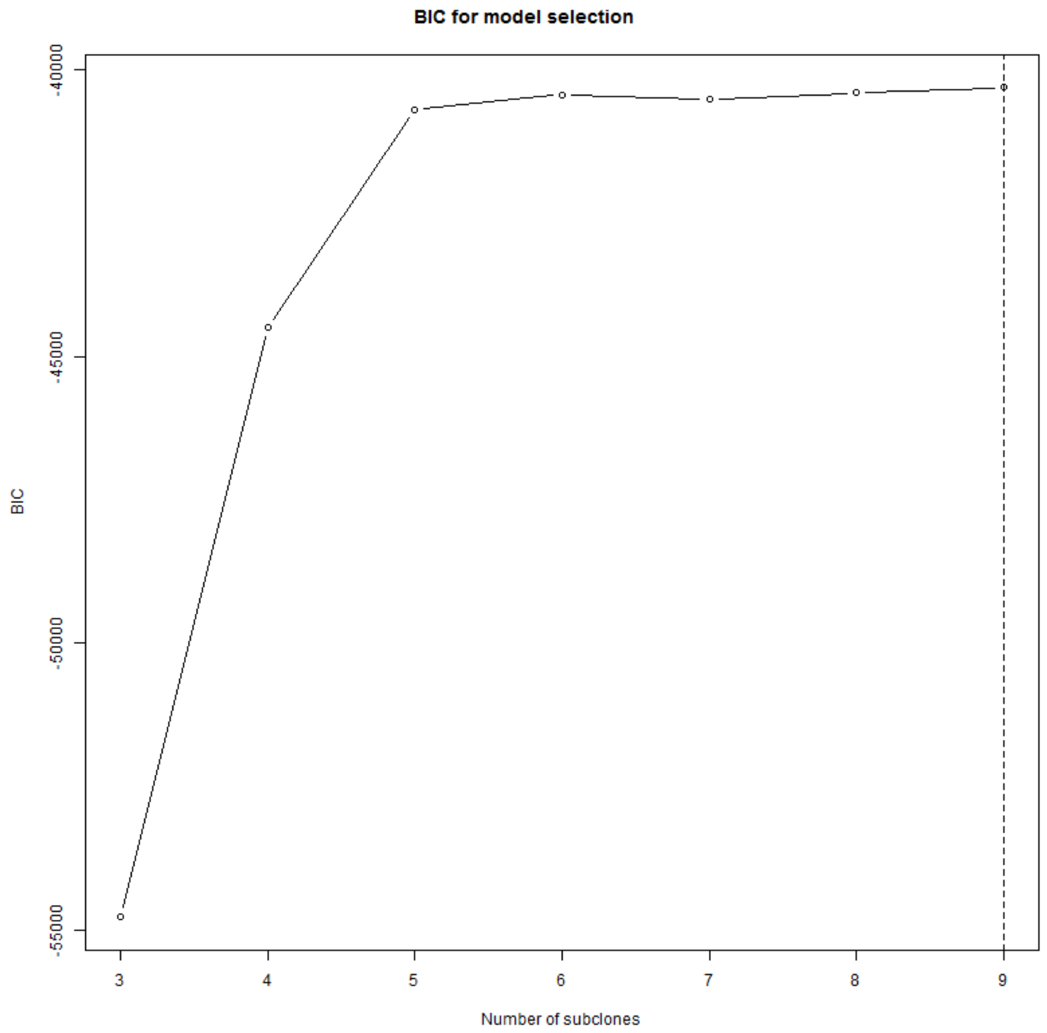

Supplemental Figure 2. BIC score of subclonal models with 3-9 clonal populations. Models with 3 to 9 clonal populations were tested by Canopy. A five-population model was selected (BIC=-40684.12), as higher-complexity models did not yield substantially higher BIC (maximal BIC=-40301.74 with 9 clonal populations). Note that germline is considered a clonal population by Canopy; therefore the selected model contains four tumor subclones.
