# Supplementary material for: Tumor heterogeneity and acquired drug resistance in FGFR2-fusion-positive cholangiocarcinoma through rapid research autopsy
Source: Cold Spring Harb Mol Case Stud. 2019 Aug;5(4):a004002. doi: 10.1101/mcs.a004002 (PMC6672025; doi:10.1101/mcs.a004002)
Supplement: Supplemental Material [file supp_mcs.a004002_Supplemental_Table1.pdf]

Sample	Percent Mutant
Negative Control	0%
Positive Control	95%
Pre Treatment	0%
Post Progression	0%
Liver #1	17.60%
Liver #2	0.30%
Liver #3	0.14%
Liver #4	0%
Liver #5	0%
Liver #6	0%
Aorta/esophagus Lymph node	0%
Right Kidney Lymph node	0%
Left Kidney Lymph node	0%

Supplemental Table 1. ddPCR for FGFR2 N549H Mutation. Droplet digital PCR (ddPCR) results for percentage of mutant present in samples.
